# Thematic Contents of Mental Imagery are Shaped by Concurrent Task-Irrelevant Music

**DOI:** 10.1177/02762366231193145

**Published:** 2023-08-09

**Authors:** Liila Taruffi, Ceren Ayyildiz, Steffen A. Herff

**Affiliations:** 1 Music Department, 3057 Durham University, Durham, UK; 2 The MARCS Institute for Brain, Behaviour and Development, 6489 Western Sydney University, Sydney, Australia

**Keywords:** mental imagery, imagination, music, affect, social dynamics, linguistic analysis

## Abstract

Imagination plays a key role in evidence-based, cognitive therapies, and recent research highlights that music – a perceptual stimulus imbued with affective and social meaning – can influence some aspects of imagination, such as vividness and emotional tone. However, little is known about music's capability to facilitate specific imagery themes that may be relevant for therapy. Here, we examine whether the quantity and quality (related to themes of affect, social dynamics, and confidence) of people's imagery is affected by the presence of task-irrelevant background music. One hundred participants imagined the continuation of a figure's journey while listening to different musical excerpts or silence. Written reports of imagined journeys underwent linguistic analysis to reveal the number of words belonging to the themes of interest. Bayesian Mixed Effects models revealed that music (*vs*. silence) led to longer reports and predicted imagery characterised by affect, social dynamics, and confidence. Implications for therapy are discussed.

Music behaviours are seemingly omnipresent across the world and throughout human history (Conard, Malina & Münzel, 2009; Cross, 2001; Merriam, 1964) to a large extent due to the pivotal role they play in affect and social dynamics. Music allows non-verbal communication, evocation and regulation of emotions during the course of the lifespan (Juslin & Laukka, 2003; Juslin & Sloboda, 2011; McFerran, Derrington & Saarikallio, 2019), and it is one of the most effective tools people use in daily life to uplift moods (Granot et al., 2021). Music-evoked emotions are mentioned among the deepest experiences people remember (Gabrielsson, 2011), and favourite music leads to intensely pleasurable states associated with the release of dopamine in the brain's mesolimbic system, as it occurs for biologically adaptive behaviours including eating and sex (Salimpoor et al., 2011; Zatorre & Salimpoor, 2013). Music making – a form of interpersonal coordinated motor behaviour – facilitates prosocial effects, such as increasing interpersonal bonding, establishing social dynamics, and strengthening group cohesion in adults and children (Hove & Risen, 2009; Kirschner & Tomasello, 2010; Kokal et al., 2011; MacRitchie et al., 2018; Rabinowitch, Cross & Burnard, 2013). Evolutionary theories have in fact contended that the adaptive value of music lies in its capability to create or maintain social bonding (McNeill, 1995; Savage et al., 2021). Intriguingly, the social functions that music taps onto are not only related to joint music making, but also encompass solitary music listening settings, in which music may act as a surrogate for an empathic friend (Schäfer, Saarikallio & Eerola, 2020; Taruffi & Koelsch, 2014), thus providing a proxy for sociality when feeling lonely like during the early stages of the COVID-19 pandemic (Groarke et al., 2022). In a similar way, solitary listening comes into play in self-enhancement processes (typically occurring in social interactions), where music may serve as an empowering resource for actively manipulating affect so that a positive and confident self-view is maintained (Elvers, 2016). For example, a listener may empathise with a singer who expresses a high sense of self-worth in their lyrics, and through this empathic process, they may adopt such a positive self-view for themselves.

While previous scientific research has investigated in depth the inherent affective and social nature of music, the study of music's influence on imagination is very recent (Herff et al., 2022; Küssner, Taruffi & Floridou, 2023; Taruffi & Küssner, 2019). However, it is worth noting that the topic of imagery has been traditionally explored in the cognitive sciences for decades (James, 1890; Kosslyn, Thompson & Ganis, 2006; Thomas, 1999). Mental imagery is broadly defined as a sensory-like experience in a modality (e.g., visual, auditory, etc) in the absence of an actual corresponding sensory input in that modality (Kosslyn, Behrmann & Jeannerod, 1995). Beyond social and emotional functions, people commonly listen to music to fantasise, empower themselves, mind wander, escape or introspect (Elvers, Fischinger & Steffens, 2018; Hays, 2006; Küssner & Eerola, 2019; North, Hargreaves & Hargreaves, 2004; Schäfer et al., 2013) and all these mental experiences are rooted in imagery processes. Schäfer et al. (2013) surveyed the literature on music functions from the past 50 years and conducted a principal component analysis on a total of 129 non-redundant musical functions. Their analysis revealed that people listen to music to achieve three core functions: self-awareness (including the imagery-based functions mentioned above), social relatedness, and arousal and mood regulation. Importantly, imagination plays a crucial role in self-regulation, health, and well-being (D’Argembeau & Van der Linden, 2006; Holmes, Arntz & Smucker, 2007), and can be altered in clinical populations (e.g., the involuntary intrusion of traumatic images is a distinctive feature of post-traumatic stress disorder; Brewin, 2014). While imagery-based therapies, such as imaginal exposure therapy, are currently a powerful and effective means for the treatment of a wide range of mental disorders (Arntz, 2012; Pearson et al., 2015), research on music's effects on imagination and how these can be harnessed to optimise therapy is still in its infancy, although initial results are promising (see next paragraph) and the relevance of this scholarship has been increasingly recognised by researchers (e.g., Küssner, Eerola & Fujioka, 2019; Küssner et al., 2023; Margulis & McAuley, 2022). Evidence from music therapy has also demonstrated the instrumental role that music-evoked mental imagery plays in the regulation of emotion, stress, and anxiety. For example, the Bonny Method of Guided Imagery and Music (GIM; Bonny, 2002) is currently considered one of the most prominent forms of receptive music therapy. It combines Western classical music and visual imagery to facilitate personal growth, relaxation, and emotional healing. GIM has been found to reduce cortisol levels (McKinney et al., 1997a), β-endorphins (McKinney et al., 1997b), as well as some side effects of chemotherapy, such as anxiety, nausea, and vomiting (Karagozoglu, Tekyasar & Yilmaz, 2013). Similarly, imagining social interactions while listening to music can facilitate the release of stress and a wide range of emotions (i.e., “catharsis” function; see Honeycutt & Harwood, 2019).

A recent study, combining an imagination task and music listening (Herff et al., 2021), demonstrated that music systematically probes a number of characteristics of imagination. Participants watched a video of a figure travelling towards a barely visible landmark and then, with closed eyes, imagined their own continuations of the journey while listening to music or during silence. Bayesian Mixed Effects models revealed that participants’ ratings of vividness, sentiment, as well imagined time passed and distance travelled, were significantly influenced by the music, and showed that aspects of these effects could be modelled through features, such as tempo (e.g., fast tempi predicted lower imagined distance and time). Dahl and colleagues (2022) adopted a production approach (i.e., producing new music stimuli by systematically manipulating a set of cues of existing music) to further explore the relationship between musical structure and visual mental imagery. By employing two original and two newly composed pop music pieces, which combined the musical and acoustical characteristics of the originals, the authors demonstrated that participants experienced comparable contents of visual mental imagery in response to pieces with similar music structure. Moreover, a content analysis of the participants’ descriptions revealed that their reported images could be categorised within the following ten themes: *nature*, *humans*, *affects*, *colours*, *places and settings*, *film*, *literal sound*, *action and movements*, *time*, and *objects*. Evidence that imagination is shaped by musical structural features also comes from studies on narratives. For example, when people imagined narratives while listening to music, they tended to imagine new events at timepoints linked to musical features, such as the entrance of a new theme (Margulis et al., 2022). Furthermore, research on mind-wandering has shown that music's emotional qualities impact thought content in a corresponding way. Specifically, participants reported more positive and empowering thoughts after listening to happy and heroic music, respectively, compared with sad music (Koelsch et al., 2019; Taruffi et al., 2017). Results on the relationship between emotion and imagery have recently been extended to ecologically valid settings such as an experimental music concert (Deil et al., 2022) and personal music listening in daily life (Taruffi, 2021). For instance, Deil et al. (2022) showed that a live concert afforded frequent visual imagery and that the concertgoers’ imagery descriptions were centred around themes (e.g., *darkness*) reflecting to a large extent the eerie tone of the music programme.

Taken together, the literature reviewed above speaks about music's capability to shape the phenomenology of imagination via different routes, such as evoked emotions, and structural and expressive properties of the music. However, most of these studies made use of subjective ratings (obtained via likert scales) of imagery content on a selected number of dimensions (e.g., content related to the past, present, or future; content related to the self or others), which has the disadvantage of imposing an already existing categorisation to the phenomenology of imagery, thereby restricting and directing participants’ answers. Most importantly, even those few studies employing participants’ free text reports of imagery (e.g., Dahl et al., 2022) did not employ a control silence condition, which is necessary to disentangle the effects of the emotions portrayed or evoked by the music from those of the music as a stimulus *per se*. To address these pitfalls, here we analysed the free text reports of 100 participants who attended a previously published imagination study (Herff et al., 2021) that required them to imagine the continuation of a journey during various music excerpts and silence. We tested a straightforward question: whether the quantity and quality of people's imaginings inspired by music would differ from the ones evoked during silence. Specifically, we hypothesised that music would trigger *more* mental imagery when compared with silence, given the recent literature pointing at the capability of music to shape imagination (e.g., Küssner et al., 2023). Furthermore, music (compared with silence) would be associated with imagery content *richer* in affect, social dynamics, and confidence, given that music is imbued with affective and social meaning (e.g., Juslin & Sloboda, 2011; Savage et al., 2021; Tarr, Launay & Dunbar, 2014), and it is often used as a tool for empowerment (Elvers et al., 2018) and to enhance motivation (see also the use of music in sport; Karageorghis & Priest, 2012).

## Method

### Participants

The sample consisted of 100 participants, who were 18 to 55 years of age (*M* = 28.3, *SD *= 9.8; 34 females, 65 males, 1 preferred not to disclose). Participants were located in Europe (88%), North America (7%), Africa (2%), South America (2%), and Asia (1%). The Musical Training subscale of the Goldsmiths Musical Sophistication Index (Gold-MSI; Müllensiefen et al., 2014) was administered to measure formal training in music (e.g., private lessons and instrumental practice). Participants’ scores ranged from 7 to 39 (out of a possible range of 7–49), with a mean score of 12.05 (*SD* = 9.73), which corresponds to the 14th–15th percentile of the data norms from Müllensiefen et al. (2014). Participants were recruited via Prolific and were reimbursed with CHF 12. They were required to be fluent in English, and be able to play back the audio. No participants reported any hearing impairments. All participants gave informed consent according to the procedures approved by the Ethics Committee of the École Polytechnique Fédérale de Lausanne and the experiment was performed in accordance with the ethical standards outlined by the Declaration of Helsinki.

### Imagination Task

**Figure 1.**
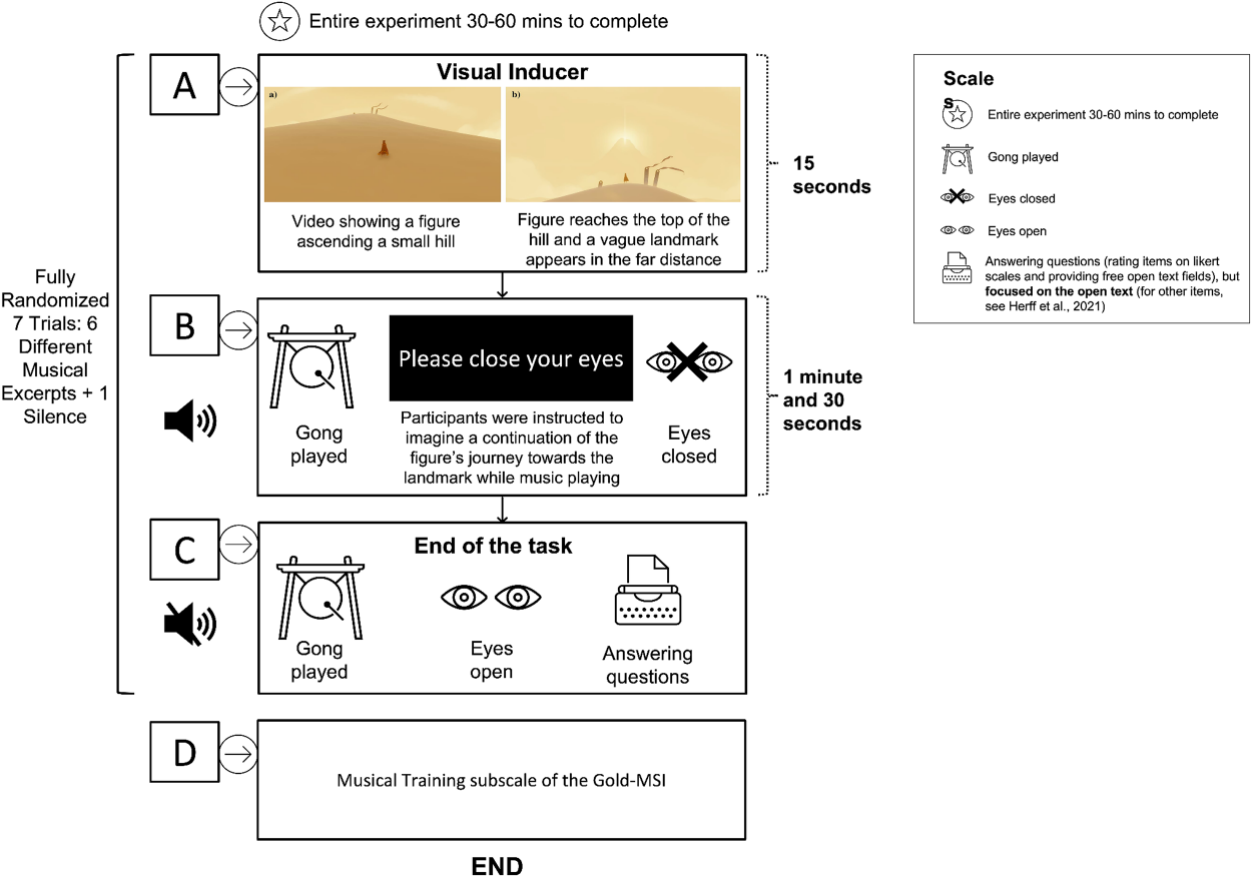
Imagination Task. A. 15-s video showing a figure ascending a small hill. Once the figure reached the top of the hill, a vague landmark appeared in the far distance. B. Participants heard a gong-sound and were instructed to close their eyes and imagine a continuation of the figure's journey towards the landmark. The imagination task lasted one minute and thirty seconds (excluding the initial visual inducer), and was accompanied by a black screen with white lettering stating, “Please close your eyes”. During each trial, participants either listened to music or a silent control condition. C. At the end of the task, the gong-sound was played again, signalling participants to open their eyes and answer a series of questions by both rating different items on likert scales and providing free open text fields. For the current study, we only focused our analysis on the open text item asking participants to describe their imagined journeys in as much detail as possible (for the other items, see Herff et al., 2021). D. Participants were asked to fill out the Musical Training subscale of the Gold-MSI.

Participants completed an imagination task in which they were first presented with a video of a figure ascending a hill and were then asked to imagine the continuation of the figure's journey while attending different auditory conditions, including six music inducers and a silent control condition. After each of the seven randomised trials, participants had to provide a free-text report describing their imagined journeys in as much detail as possible. Details of the imagination task are illustrated in Figure 1. The visual inducer was taken from the video game “Journey” with written permission of Jenova Chen, CEO of ThatGameCompany (https://thatgamecompany.com). The entire experiment took between 30 and 60 min to complete, largely depending on the depth of detail provided by the participants in the open-ended description of the imagined journey.

### Music Stimuli

The six music stimuli used in this experiment were excerpts of three classical music and jazz pieces (J. S. Bach, Chorale *O Haupt voll Blut und Wunden* from Matthäus Passion BWV244; C. Debussy, *Tarantelle Styrienne* L. 69; R. Rodgers, *My Favorite Things*). Instrumental music was chosen over music with lyrics to keep the design as simple as possible, since lyrics have been found to induce effects on listeners’ emotion and imagery that are difficult to separate from those of music alone (Barradas & Sakka, 2022; Brattico et al., 2011; Susino & Schubert, 2019). Stimuli had the same length (1 min and 30 s) and their loudness levels were all normalised. Each piece was presented in two renditions (illustrated in Table 1, along with their abbreviations), which were identical, or very similar, in terms of music structure, but differed in performers or instrumentation. The differences between the two renditions of the jazz piece *My Favorite Things* were more substantial, and, besides instrumentation, also encompassed local harmonic, rhythmic and melodic features, and large-scale musical structure. This approach allowed us to get specific insights into the musical properties that are linked to imagery content.

**Table 1.** Music Stimuli Selected for the Study.

Title	Composed by	Conducted by	Performed by	Year	Abbreviation
O Haupt voll Blut und Wunden	J. S. Bach	J. E. Gardiner	Monteverdi Choir and the English Baroque Soloists	1989	BachG
O Haupt voll Blut und Wunden	J. S. Bach	W. Furtwängler	Wiener Singakademie and Wiener Philharmoniker	1954	BachF
Tarantelle Styrienne	C. Debussy	/	J.-Y. Thibaudet (piano rendition)	2000	DebussyT
Tarantelle Styrienne	C. Debussy	L. Slatkin (orchestrated by M. Ravel)	Orchestre National de Lyon	2016	DebussyR
My Favorite Things	R. Rodgers	*/*	The John Coltrane Quartet	1961	MFTC
My Favorite Things	R. Rodgers	*/*	B. Mehldau	2010	MFTM

### Linguistic Analysis and Statistical Approach

To test whether music and silence would differ in quantity and quality of evoked imagery, we preprocessed participants’ free text responses, conducted linguistic analysis to identify the relative proportions of the selected themes, and statistically assessed the differences between music and silence for each theme.

Participants’ responses were preprocessed by removing any reference to the imagination task (e.g., “I imagined”, “after the gong sound”, “for this journey”) or comment about the music (e.g., “I did not like the music”, “the music made me feel”), thus retaining only the contents of the imagined journeys.

Reports were then analysed using Linguistic Inquiry and Word Count (LIWC; Pennebaker et al., 2015). LIWC is a prominent tool for analysing text data, which performs automated categorisation of words into themes, using a set of internal dictionaries that capture psychological phenomena related to cognition, affect, personal concerns, and social dimensions. LIWC has been extensively validated across various research domains and shown to be a reliable tool for text analysis (e.g., Cohn, Mehl & Pennebaker, 2004; Newman et al., 2003); furthermore, LIWC better captures emotional expression compared with other existing programs for automated text analysis (e.g., Bantum & Owen, 2009). In music research, LIWC has been successfully employed to reveal phenomenological properties of autobiographical memories (e.g., Janata et al., 2007) as well as mind-wandering (Taruffi et al., 2017). Here, we specifically focused on the LIWC variables named “Emotional Tone” and “Clout” as well as the standard “Word Count”. “Emotional Tone” measures both positive (e.g., happy, good) and negative (e.g., kill, guilty) emotion words, with high scores reflecting an overall positive emotional tone (for an example of an application of this index, see Cohn et al., 2004). “Clout” indicates the extent to which people write with a sense of confidence and certainty, and use pronouns that are more others-focused (e.g., they, we) and social words (e.g., help, fellow) (for an example of an application of this index, see Fox & Royne Stafford, 2021). Loadings on the three variables of interest, consisting of percentages of total words falling into the selected dictionaries, were further analysed using Bayesian Mixed Effects models.

The models predicted independently “Word Count”, “Emotional Tone”, and “Clout”, trial-wise, either based on whether a given trial had music or not (for general music *vs.* silence comparison), or based on the precise sound condition played (Silence, BachF, BachG, DebussyR, DebussyT, MFTC, MFTM), whilst accounting for participant and trial random effects. Each model ran on 4 chains, with 10000 iterations, 1000 warm-ups, and was initialised with zeros. All continuous variables were normalised to have a mean of zero and a standard deviation of 1. The models were given a weakly informative prior in the form of a t-distribution with mean 0, standard deviation of 1, and 3 degrees of freedom. The prior as well as standardisation approach is commonly used in the auditory as well as music cognition literature (Beveridge, Cano & Herff, 2022; Cecchetti, Herff & Rohrmeier, 2022; Herff et al., 2020, 2023; MacRitchie et al., 2020; Smit et al., 2022). We reported the model's effect estimates (*β*), the error of these estimates (*EEβ*), and the evidence ratio of a directed hypothesis test that a given musical condition is predictive of an increase in a specific theme or word count (*Odds*(*β* > 0). For convenience, we indicated effects that can be considered “significant” under an alpha level of 5% with * (i.e., evidence ratio ≥ 19; see Milne & Herff, 2020).

## Results

### Word Count

**Figure 2.**
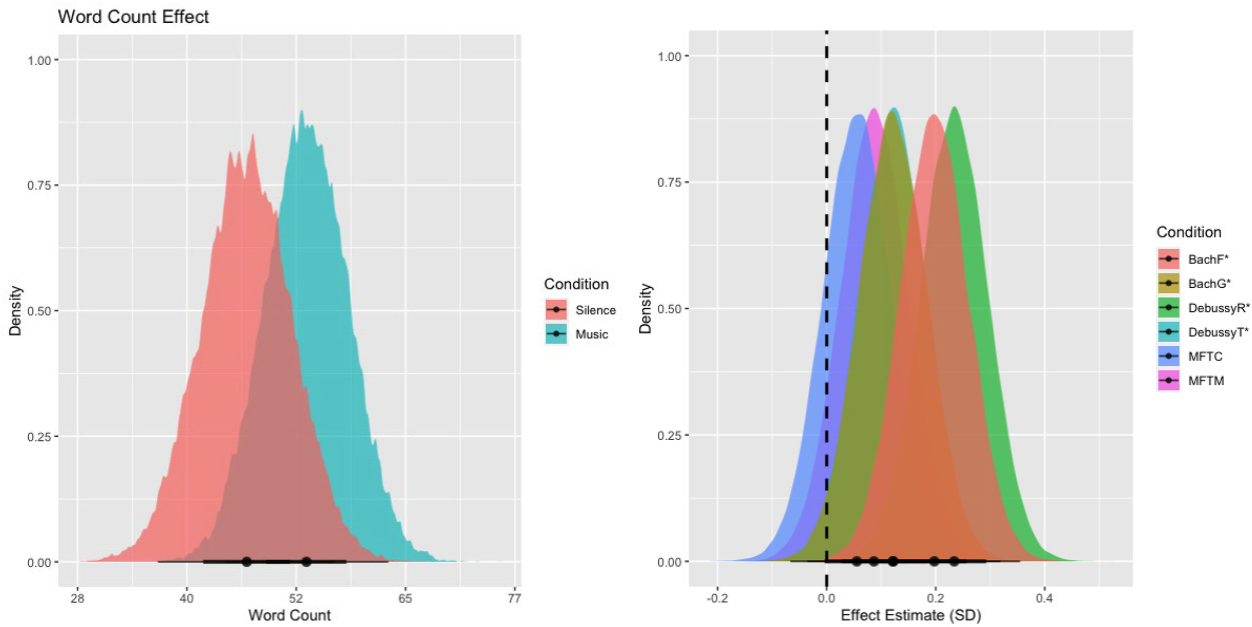
The left panel shows the model's linear posterior predictions for word count in the music (red) and silent (blue) conditions. The right panel shows the posterior distributions of the coefficients for the individual music pieces. Overall (left) —as well as in four out of six music conditions when tested individually (right)— we find that music is predictive of higher word counts. Only the two renditions of *My Favorite Things* (MFTC, MFTM) do not provide compelling evidence for this effect, however, they are still descriptively predicting more words than the silence condition. The reported evidence ratios refer to the ratio of evidence (mass under the curve) of the posterior distribution that lies to the right of the dashed vertical zero line compared to the evidence that lies to the left of the dashed zero line.

As seen in Figure 2 (left), we observed strong evidence for greater word counts (14% more on average) in music (*M* = 50.16, *SE* = 5.02) compared to silent (*M* = 46.75, *SE* = 4.19) imagination trials (*β* = 0.14, *EEβ* = 0.05, *Odds*(*β* > 0) = 438*). When broken down by music piece, all pieces showed descriptively more words than the silent control condition, with strong evidence for this effect in both renditions of *O Haupt voll Blut und Wunden* (BachF: *β* = 0.20, *EEβ* = 0.06, *Odds*(*β* > 0) = 2399*; BachG: *β* = 0.12, *EEβ* = 0.06, *Odds*(*β* > 0) = 37.63*) and both renditions of *Tarantelle Styrienne* (DebussyR: *β* = 0.23, *EEβ* = 0.06, *Odds*(*β* > 0) =7199*; DebussyT: *β* = 0.12, *EEβ* = 0.06, *Odds*(*β* > 0) = 40.38*); only the two renditions of *My Favorite Things* did not show strong evidence for this effect (MFTC: *β* = 0.06, *EEβ* = 0.06, *Odds*(*β* > 0) = 4.35; MFTM: *β* = 0.09, *EEβ* = 0.06, *Odds*(*β* > 0) = 11.16), which can be seen in Figure 2 (right).

Examples of text reports of the participants’ imagined journeys, scoring low/medium/high on the “Word Count” variable for both music and silence conditions, are provided in Table 2.

**Table 2.** Examples of Text Reports of Imagined Journeys, Scoring Low/Medium/High on the LIWC Variables of Interest, for Both Music and Silence.

LIWC Variable	Low Score	Medium Score	High Score
**Emotional Tone**			
Music	“I was walking through a desert filled with strange machinery. The whole thing was like some big factory. Everything was automated and seemed to have a life of its own. I wondered what these machines were for, but it was hard for me to say.” (MFTC)	“I came down from the little mountain and magically everything in front of me changed to a meadow. There were lakes around me, lots of flowers, animals. I sat near the lake and ate some food and drank water. I stood up and kept going. The animals were coming to me, they wanted me to pet them. It was very fun.” (DebussyR)	“I realized that life was good. I was extremely happy, and I wanted to rush until I reached the highest point of the second mountain. When I got there, I was able to see the sea and the waves. Simply amazing.” (MFTC)
Silence	“It was night and there was snow everywhere. I was freezing and I felt tired. I kept waking until I found a place where I decided to stop and sleep there. It was protected from winds there and I was trying to camp there [when I heard the gong].”	“I was on the top of the hill, I took a moment to breathe, then I decided to continue my walk and come down from the mountain, I arrived in a kind of big sandy desert surrounded by fences. I felt calm, the wind blowing my face, I was looking around [when a bell rang].”	“Below a field full of flowers dancing nymphs dwarfs he stayed and danced with them happily then small lake below, he started climbing the rope to a high mountain. At the top there was a life giving lake in which he took a bath. He came down and saw a small forest.”
**Clout**			
Music	“I was highly motivated to reach the peak of the highest mountain, so I was doing everything I could to reach it as soon as possible. When I arrived there, I was simply amazed by what I was seeing.” (DebussyT)	“I walked for several hours until I came across a building in the desert, surrounded by palm trees. I knocked on the door and an old bearded man answered. I explained that I was travelling to the mountain and asked if I could stay for the night. He agreed with an open-heart. As we walked through the building, he explained that this was a monastery, he was a monk, lived here with three others and they went by the name: Desert Fathers.” (BachF)	“The character continued to climb down the hill. Initially [I imagined] good weather and people dancing around the main character. However, [as the music got more agitated, I imagined] the character going inside a dark forest and being chased by something or someone. And he was trying to escape.” (DebussyT)
Silence	“I took a time to wonder why I was alone in that place. Then I thought that it would be a great idea to start my journey up to the second peak, as there I could possibly see someone. I started the journey in a normal space, without rushing.”	“Very cold and windy. It was just me and the horse, it felt like time was going by fast, the aim was to arrive on the hill as soon as possible.”	“I started my journey to the top of the mountain. First day of the journey was pretty peaceful. I had to walk slowly, because the sun on the desert is extremely hot. On the next day afternoon I was attacked by giant desert ants. There was too many of them. I almost died. Suddenly some masked man ran out of the nearest rock and started killing these ants. After he helped me, he took his mask off. It was a man I met in the city just before my journey to the mountain. I told him about my plans. Now he saved my life and thinks that he deserves a part of the treasure from the top of the mountain. Last day was the hardest. We lost our water. Hank (the name of the man) passed out, so I grabbed him, and carried him by the top of the mountain. At the end of the journey Hank woke up, and we both saw a beautiful sight from the top, and a bunch of golden cases.”
**Word Count**			
Music	“I went down the first mountain and I arrived at a small village, where I found a river and I refreshed my face.” (MFTM)	“I ascend the small hill. At the top I can see the mountain in the distance in front of me. It's probably around a kilometre away and it doesn't seem too big. I close my eyes and when I reopen them I find myself at the foot of the mountain. It's not too steep. The ground is covered in dirt and rocks and around me I can only see some bushes, but with only their branches remaining. I start climbing up the mountain. Here and there some bigger rocks block my way, but it's nothing I can't climb over. The scenery doesn't change. The sky above me is clear and birds are flying in flocks. I can faintly hear cars in the distance, but when I look around me, I can't see any roads in the distance. After around a kilometre I reached the top. It is a flat platform with a couple of trees and a bench on the edge of it. I reach the bench and decide to rest on it for a while. The view is amazing. I can see the desert slightly to the left and to the right there are green meadows and forests. Beyond them another mountain rises, even bigger than the one that I have already climbed. Behind it, I can see the sun settling and the sky has beautiful colours of pink and orange hues.” (BachG)	"Ah, our traveler has reached the top of the hill, to see one of his biggest challenges yet, the Shade of the mountain was big enough to give any climber some shivers, but our traveler thought naught of such triviality, as he had to do this, otherwise his position at the Throne would be in danger. He had a time limit, so he decided to rush it as much as he could. Decided to slide down towards the base of the mountain which saved him some time, alas, walking would have been the best idea to conserve stamina but that never came across his thoughts. Before he even started to climb he saw several spots that looked like some sort of caverns, and made his path with those in mind to rest, otherwise this challenge would be rather impossible, the Traveler, daring everything obstacle he could, climbed faster than everyone should do, which made him waste his first day, really quick. He got to the first cavern and rested for around what he would thought it would be: 1 day and 3 h, with such Stamina and the sun starting to rise, he kept on climbing, easily spent another 10 h climbing, just to find out one of the cavern was a nest for birds, so he couldn't really rest there properly. 5 days have gone by, our traveler, amidst of all the things that he came across, is now, by the half point of the mountain, such achievement was done by nobody, yet he amazed himself as he saw that the half of the mountain was marked with a message which he couldn't read due to the rush of the challenge. He fought birds, birds… and the wind, there was actually no real threat to him during the climb aside of him being exhausted, on the 9th day, he spent 25 min sitting on what would be almost the end of his challenge, thinking if people felt happy, if the kingdom was doing well, if everyone was safe, he really cared for his people, as he kept on thinking about it, he just gained the last bit of energy he needed, he wouldn't leave the challenge. The top was achieved, and on it, he was being waited for a member of his committee. Which granted him the recognition to stay on the throne in the future for such dedication, committed only to save the people from his kingdom, to live from such a harsh future with a possible tyrant.” (BachG)
Silence	“I walked through the desert for many kilometres. I saw some temple in the distance. Halfway through the trip I hurt my leg.”	“As the sun rose, the man in his Journey would see another mountain higher than the hill he just climbed…, nevertheless on his way down he could simply slide, hence making it to the middle point of the mountain way easier, but getting up there would be trouble due to having to cross the break point of it, Canyon as would people usually call it. It was an extensive walk, seeming almost endless until the sun that wouldn't really shine in the Canyon, started to guide him out, as he walked out of it, he started to notice he was feeling exhausted, it felt like a 9 h and 31 min walk approximately and it was due that he never walked on a plain ground, he was climbing under the canyon. His goal to reach the top of the mountain wasn't very far from him, about a 1.8 km climb to reach the goal and check what was waiting for him at the end.”	/

*Note.* Scores: low: <  33%; medium: > 33% and < 66%; high:  > 66%. No example for Word Count/High Score for the silence condition is provided because there was not any description fulfilling the required word count.

### Emotional Tone

**Figure 3.**
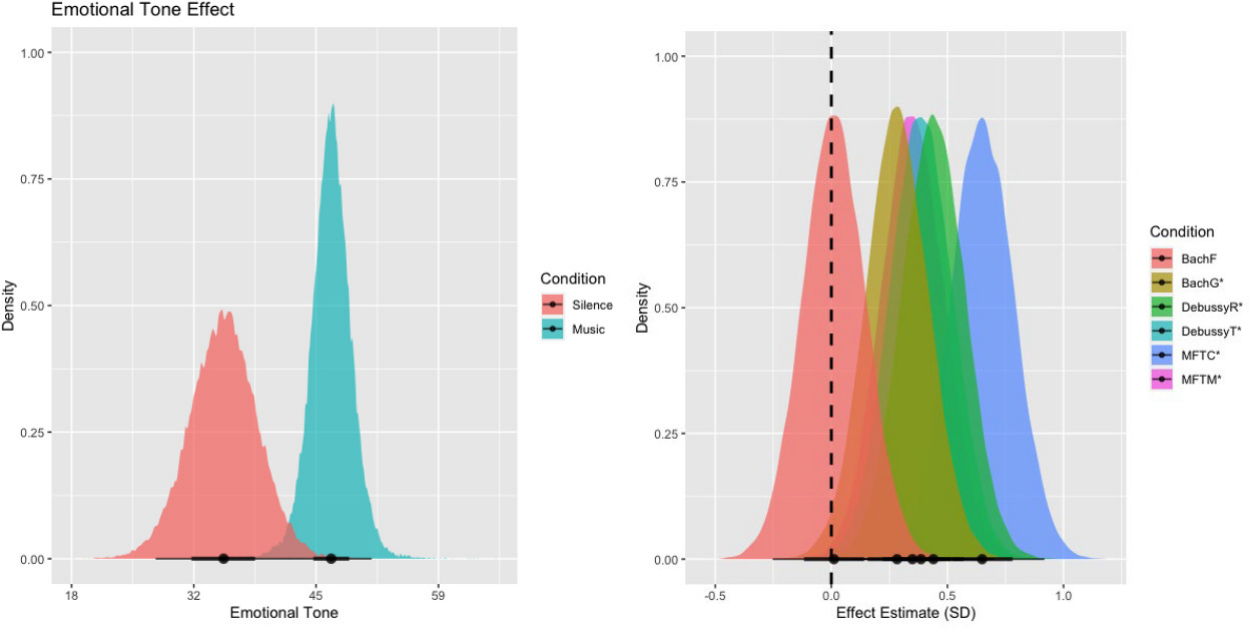
The left panel shows the model's linear posterior predictions for emotional tone in the music (red) and silent (blue) conditions. The right panel shows the posterior distributions of the coefficients for the individual music pieces. Overall (left)—as well as in five out of six music conditions when tested individually (right)— we find that music is predictive of more emotional language. Only one rendition of *O Haupt voll Blut und Wunden* (BachF), did not provide compelling evidence for this effect. The reported evidence ratios refer to the ratio of evidence (mass under the curve) of the posterior distribution that lies to the right of the dashed vertical zero line compared to the evidence that lies to the left of the dashed line.

We observed strong evidence for more emotional language (34% more on average) in music (*M* = 46.93, *SE* = 3.46) compared to silent (*M* = 34.99, *SE* = 3.07) imagination trials (*β* = 0.35, *EEβ* = 0.11, *Odds*(*β* > 0) = 1799*), as seen in Figure 3 (left). When broken down by music piece, as depicted in Figure 3 (right), all pieces showed descriptively more emotional language than the silent control condition, with strong evidence for this effect in both renditions of *Tarantelle Styrienne* (DebussyR: *β* = 0.44, *EEβ* = 0.14, *Odds*(*β* >0) = 1999*; DebussyT: *β* = 0.39, *EEβ* = 0.13, *Odds*(*β* > 0) = 417.60*), both renditions of *My Favorite Things* (MFTC: *β* = 0.65, *EEβ* = 0.14, *Odds*(*β* > 0) = 9999*; MFTM: *β* = 0.35, *EEβ* = 0.14, *Odds*(*β* > 0) = 218.51*), but only one rendition of *O Haupt voll Blut und Wunden* (BachF: *β* = 0.01, *EEβ* = 0.14, *Odds*(*β* > 0) = 1.14; BachG: *β* = 0.28, *EEβ* = 0.14, *Odds*(*β* > 0) = 54.99*).

Examples of text reports of the participants’ imagined journeys, scoring low/medium/high on the “Emotional Tone” variable for both music and silence conditions, are provided in Table 2.

### Clout

**Figure 4.**
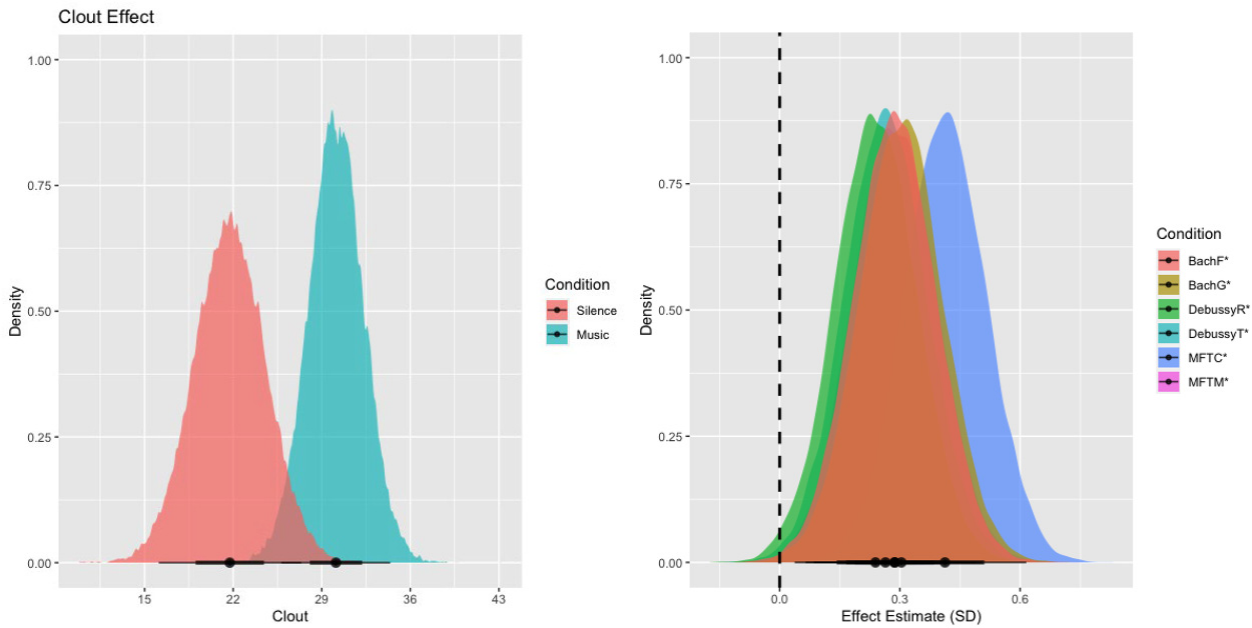
The left panel shows the model's linear posterior predictions for clout in the imagined content generated in the music (red) and silent (blue) conditions. The right panel shows the posterior distributions of the coefficients for the individual music pieces. Overall (left)—as well as in all six music conditions when tested individually (right)— we find that music is predictive of more language related to social dynamics and confidence. The reported evidence ratios refer to the ratio of evidence (mass under the curve) of the posterior distribution that lies to the right of the dashed vertical zero line compared to the evidence that lies to the left of the dashed line.

We observed strong evidence that music (*M* = 30.15, *SE* = 2.79), compared to silence (*M* = 21.90, *SE* = 2.56), predicts more language related to social dynamics and confidence (37% more on average, *β* = 0.3, *EEβ* = 0.08, *Odds*(*β* > 0) = 8999*), as seen in Figure 4 (left). When broken down by music piece, as shown in Figure 4 (right), all pieces exhibited strong evidence for more language referring to social dynamics and confidence than the silent control condition (DebussyR: *β* = 0.24, *EEβ* = 0.1, *Odds*(*β* > 0) = 102.45*; DebussyT: *β* = 0.26, *EEβ* = 0.1, *Odds*(*β* > 0) = 208.3*; MFTC: *β* = 0.41, *EEβ* = 0.1, *Odds*(*β* > 0) = 9999*; MFTM: *β* = 0.29, *EEβ* = 0.1, *Odds*(*β* > 0) = 432.73*; BachF: *β* = 0.30, *EEβ* = 0.1, *Odds*(*β* > 0) = 386.1*; BachG: *β* = 0.30, *EEβ* = 0.1, *Odds*(*β* > 0) = 609.17*).

This finding was further supported by manually annotated analysis, showing strong evidence for more imagined social interactions in music (*M *= 0.36, *SE *= 0.02) compared to silent (*M *= 0.17, *SE *= 0.04) condition (*β* = 1.47, *EEβ* = 0.34, *Odds*(*β* > 0) > 9999*, see supplemental material).

Examples of text reports of the participants’ imagined journeys, scoring low/medium/high on the “Clout” variable for both music and silence conditions, are provided in Table 2.

## Discussion

In this study, we tested whether listening to music (*vs.* silence) triggers more mental imagery, and whether such music-evoked imagings feature themes related to affect, social dynamics and confidence. Our findings support these hypotheses, with all the themes of interest, as well as word count, exhibiting significant effects of music.

Specifically, we corroborate previous findings from our reference study (Herff et al., 2021), which showed that music (*vs.* silence) led to increased affect, by performing a sentiment analysis of the qualitative data with the software NLTK (Loper & Bird, 2002). Here we obtained the same result (i.e., LIWC variable “Emotional Tone”), but using another analytic tool, trained on a different corpus, thereby demonstrating the high reliability of this finding. The finding that music modulates emotional aspects of imagery is in line with other prior work (Koelsch et al., 2019; Taruffi et al., 2017); however, none of these studies compared music with a silence control condition, which is necessary to identify music-specific effects. Although affective characteristics of evoked imagery varied according to the music stimuli, they overall tended to be positively valenced, echoing the results of previous music studies exploring evoked emotions and thoughts in daily life (e.g., Juslin & Laukka, 2004; Taruffi, 2021). Our analysis also revealed that the imagery reports provided in response to music were overall longer than the ones provided during silence (i.e, LIWC variable “Word Count”), suggesting that music stimulates richer stories in one's mind. Furthermore, music-evoked imagery reports exhibited a usage of words reflecting social dynamics and confidence (i.e., LIWC variable “Clout”), with the most common social reference being “people” (see supplemental material). This finding is in line with previous research showing that music elicits thought and imagery contents characterised by social elements. For example, Taruffi et al. (2017) reported that listening to happy music is associated with imaging people dancing; and “people” and “humans” were identified as imagery categories (among others) in response to experimental and pop music (Deil et al., 2022; Dahl et al., 2022). With regard to the theme of confidence, our results align well with the finding that heroic music is associated with positive, exciting, and motivating thoughts (Koelsch et al., 2019).

This study presents novel evidence that the phenomenology of imagery evoked during music differs from that evoked during silence. We suggest that music's intrinsic emotional and social nature projects onto the contents of imagery, providing multidimensional material to fuel people's imagined stories. However, to precisely determine *why* music is capable of doing so and *whether* this capability is “unique” to music, future research should implement causal designs and compare music with other perceptual cues (e.g., taste) or art forms (e.g., visual art). Furthermore, our study adopts a characterisation of the contents of imagery based on the language used by participants to report their imagined journeys, thereby constituting an indirect assessment of the actual imagery processes and yielding no precise information about the modality (e.g., visual, auditory, kinaesthetic) with which such imagings occur (Küssner & Taruffi, 2023). For this reason, future research might consider complementing rich qualitative data with covert or indirect measures, such as fMRI and drawing tasks.

Our large sample of participants showed a wide spread of age and culture, however, predominantly originated from European countries (88%) and North America (7%). Our stimuli ranged from Western classical music to jazz, but generally adhered to expectations formed by Western enculturated listeners. Consequently, we expect our findings to generalise to Western enculturated listeners, listening to familiar types of music. However, there is still a need for cross-cultural investigations, unraveling the intricate connections between diverse musical traditions across the globe and imagery, as well as explorations of potential age effects, by sampling, for example, older populations of music listeners. Furthermore, the majority of studies on music and mental imagery, including the present research, have utilised instrumental classical music. Nevertheless, music with lyrics and genres other than classical music are listened to worldwide. Therefore, it will be pivotal for future work to consider music with lyrics and a wider variety of genres.

Importantly, our findings pinpoint music as a powerful means for assisting imagery-based therapies (e.g., Pearson et al., 2015). Imagining a past traumatic experience followed by a guided intervention that alters the outcome of the past event from a negative to a positive one has been shown to be an effective treatment in a wide range of conditions such as post-traumatic stress disorder, obsessive-compulsive disorder, and affective and personality disorders (Arntz, 2012). The capability of music to enhance imagery could be harnessed to optimise such imagery-based therapies and patients who experience difficulties in their imagery abilities might benefit from listening to music during therapy. Furthermore, a therapist could use music to increase confidence levels in their patients, or to regulate emotional qualities of imagery, especially when working on issues of affective nature. In particular, music therapists, who already utilise the emotional power of music to modulate clients’ stress and relaxation levels, could harness similar psychological mechanisms to guide mental imagery. For example, the Incremental Sound Organiser principle of music therapy allows for shifting between different emotional states by listening to a medley of music (Honeycutt & Harwood, 2019). This approach could in turn be employed to induce imagery or imagined interactions (Honeycutt, 2003) with emotional connotations, enabling therapists to address trauma and relationship issues with their clients. Certainly, further research is necessary to study in detail how individual differences in imagery and music interact as well as how different types of music map onto imagery contents to inform and guide the selection of music for therapy. Thus, the complexity and clinical relevance of this topic warrant greater future investigation.

## Conclusions

Despite people commonly listen to music to fantasise, mind wander, escape or introspect, not much is known about the impact of music on the phenomenology of such imagery-based mental experiences. By carrying out a linguistic analysis of participants’ written reports of their imagined stories while attending to different musical excerpts and silence conditions, our results show that music can shape affective and social aspects of imagery, themes related to confidence, and can stimulate overall richer imagined stories in one's mind. Given that mental imagery is an integral part to cognitive therapies, this study highlights the importance of music as a tool to direct the contents of imagination.

## Supplemental Material

sj-docx-1-ica-10.1177_02762366231193145 - Supplemental material for Thematic Contents of Mental Imagery are Shaped by Concurrent Task-Irrelevant Music Click here for additional data file. Supplemental material, sj-docx-1-ica-10.1177_02762366231193145 for Thematic Contents of Mental Imagery are Shaped by Concurrent Task-Irrelevant Music by Liila Taruffi, Ceren Ayyildiz and Steffen A. Herff in Imagination, Cognition and Personality
