# Supplementary material for: Thematic Contents of Mental Imagery are Shaped by Concurrent Task-Irrelevant Music
Source: Imagin Cogn Pers. 2023 Aug 9;43(2):169–92. doi: 10.1177/02762366231193145 (PMC10620066; doi:10.1177/02762366231193145)
Supplement: sj-docx-1-ica-10.1177_02762366231193145 - Supplemental material for Thematic Contents of Mental Imagery are Shaped by Concurrent Task-Irrelevant Music [file sj-docx-1-ica-10.1177_02762366231193145.docx]

**Supplementary Material**

**Manual data coding for social interaction**

The open-ended descriptions of the imagined journey were analysed using manual data coding to compare the effects of music and silence on social interaction (*see* Table S1 for social interaction variables and their corresponding description given to the annotator). To avoid coding biases, the annotator was not provided with the condition (silence *vs.* music) under which the free-format responses were obtained.

There is strong evidence for more social interaction during music (*M* = 0.36, *SE* = 0.02) compared with silent (*M* = 0.17, *SE* = 0.04) imagination conditions (*β* = 1.47, *EEβ* = 0.34, *Odds*(*β* > 0) > 9999*), as seen in Figure S1. When the first-person language usage was compared with third-person language usage during music (*M* = 1.33, *SE* = 0.7) and silence (*M* = 1.30, *SE* = 0.7) trials, there was no significant difference between the two conditions (*β* = 0.46, *EEβ* = 0.69, *Odds*(*β* > 0) = 2.97). We also assessed social interaction through breaking it down to human and non-human social interaction (Figure S2). There was no significant effect on music (*M* = 0.22, *SE* = 0.04) compared with silent (*M* = 0.09, *SE* = 0.03) conditions (*β* = 0.46, *EEβ* = 0.69, *Odds*(*β* > 0) = 2.97).

**Frequency of nouns and descriptive verbs**

We also examined the usages of individual words across all music-evoked imagined journeys. The most frequently used (above 20 counts) nouns and descriptive verbs -ordered by their frequency, with counts listed in brackets- were as follows (plurals and conceptually equivalent words, e.g., path, footpath, pathway, were grouped together): mountain(s) [574]; walk(s/ed/ing) [290]; see(s/saw/ing) [220]; top, peak [209]; feel(s/felt/ing) [173]; go(es/went/ing) [164]; reach(es/ed/ing) [148]; hill(s) [147]; journey, trip [137]; start(s/ed/ing) [136]; imagine(s/d/ing) [129]; sand [117]; climb(s/ed/ing) [114]; city(ies), town(s), village(s) [101]; character, figure, traveller [93]; desert(s) [93]; find(s/found/ing) [88]; day(s) [83]; make(s/made/ing) [80]; keep(s/kept/ing) [78]; people [78]; take(s/took/ing) [78]; music [77]; way [74]; look(s/ed/ing) [68]; run(s/ing) [63]; come(s/came/ing) [52]; stop(s/ed/ing) [52]; travel(s/ed/ing) [52]; tree(s) [49]; end [48]; sun(s) [47]; think(s/thought/ing) [47]; night(s) [46]; fly(ed/ing) [45]; forest(s) [44]; know(s/knew/ing) [44]; side(s) [44]; place(s) [43]; path(s), footpath, pathway [42]; back [40]; bird(s) [40]; distance [38]; water(s) [38]; fall(s/fell/ing) [37]; continue(s/ed/ing) [36]; wind(s) [36]; imagination [35]; man, woman [34]; try(ies/ied/ing) [34]; animal(s) [33]; eye(s) [33]; decide(s/ed/ing) [32]; cave(s) [30]; foot(feet) [30]; hour(s) [30]; rest(s/ed/ing) [30]; destination [29]; sky(ies) [29]; dance(s/d/ing) [28]; ground [28]; view(s) [28]; dune(s) [28]; hero, heroine [27]; life [26]; river(s) [25]; rock(s) [25]; air [24]; light(s) [24]; fun [23]; horse(s) [23]; base [22]; world [22]; friend(s) [21]; landscape, scenery [21]; lake(s) [20].

Table S1. *Social Interaction Variables and Their Corresponding Description for Coding.*

| **Variables** | **Coding Description** |
| --- | --- |
| **SocialInteraction** | Codes whether (0 = No; 1 = Yes)  The described imagined episode contains any (voluntary or involuntary) communicative, cooperative, or competitive behaviour or situation between two or more entities, capable of such behaviour. Such entities could be humans, animals, fantastic creatures, or robots, if they exhibit an aforementioned behaviour or are part of an aforementioned situation. |
| **HumanSocialInteraction** | Codes whether (0 = No; 1 = Yes) SocialInteraction = 1 AND the behaviour or situation contains 2 or more humans. We assume that the figure shown in the visual inducers is considered human, even if only described as a figure. |
| **NonHumanSocialinteraction** | Codes whether (0 = No, 1 = Yes) SocialInteraction = 1 AND the behaviour or situations contains 1 or more non-human. A non-human could be an animal, a fantastic creature, or robot, if they exhibit social behaviour or are part of a social situation. |
| **Perspective** | Codes the narrative perspective the participants uses when describing or referring to the figure shown in the initial visual inducer video (1 = First Person, i.e., 'I'; 3 = Third person, e.g., the figure, the person, she/he/it/they, the girl, the boy, the traveler etc.). |


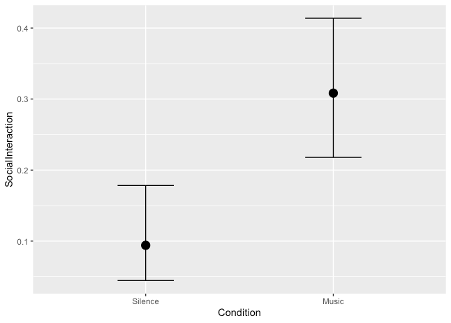


*Figure S1.* Predicted social interaction prevalence in silence and music conditions.


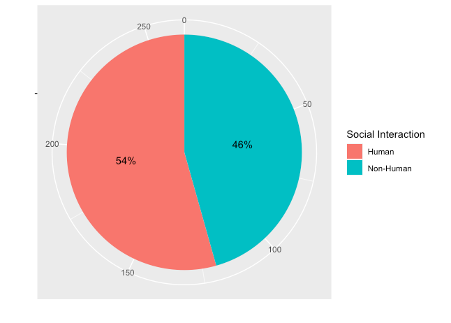


*Figure S2.* Proportion of human and non-human social interactions.
